# ecPICK: A deep learning-enabled spatial diagnostic platform for direct ecDNA identification and clinical prognosis across pan-cancer histopathology

**DOI:** 10.7150/thno.134316

**Published:** 2026-05-25

**Authors:** Xue-Ting Zhen, Zhen Yang, Lu-Ning Qin, Yun-Long Zhao, Lu Chen, Ming Gao, Tao Sun, Heng Zhang

**Affiliations:** 1 Tianjin Union Medical Center, The First Affiliated Hospital of Nankai University, College of Pharmacy and State Key Laboratory of Medicinal Chemical Biology, Nankai University, Tianjin 300350, China; 2 Department of Oncology, The Institute of Translational Medicine, Tianjin Cancer Institute of Integrative Traditional Chinese and Western Medicine, State Key Laboratory of Neurology and Oncology Drug Development, Tianjin Union Medical Center, The First Affiliated Hospital of Nankai University, Nankai University, Tianjin 300121, China.; 3 Department of Hepatobiliary Cancer, Liver cancer research center, Tianjin Medical University Cancer Institute and Hospital, National Clinical Research Center for Cancer, Tianjin Key Laboratory of Digestive Cancer, Tianjin's Clinical Research Center for Cancer, Tianjin, 300060, China.; 4 Department of Thyroid and Breast Surgery, The Institute of Translational Medicine, Tianjin Cancer Institute of Integrative Traditional Chinese and Western Medicine, Tianjin Union Medical Center, The First Affiliated Hospital of Nankai University, Nankai University, Tianjin 300121, China.

**Keywords:** ecDNA, deep learning, digital pathology, artificial intelligence, spatial transcriptomics, tumor microenvironment, spatial diagnostics

## Abstract

**Rationale:**

Extrachromosomal DNA (ecDNA) is an important driver of oncogene amplification and drug resistance; however, its clinical assessment is constrained by the high costs of sequencing and lack of spatial resolution in conventional assays. Thus, a cost-effective, clinically translatable platform is required for ecDNA quantification and localization using routine pathological samples.

**Methods:**

We developed the deep learning framework ecPICK that identifies and localizes ecDNA in routine H&E-stained whole-slide images. The model was trained and tested using 4,280 images representing 20 different cancers. Its diagnostic efficacy was evaluated by area under the curve (AUC) analysis, and its spatial accuracy was verified via fluorescent in situ hybridization (FISH). In addition, the tumor microenvironment associated with ecDNA was examined by combining ecPICK with spatial transcriptomics.

**Results:**

ecPICK showed strong agreement with FISH-validated ecDNA levels (R^2^ = 0.85), with a strong pan-cancer AUC of 0.789. Among the clinical cohorts, ecPICK identified ecDNA as an independent prognostic predictor beyond detection. Based on spatial research, ecDNA-rich areas preserve a unique microenvironment marked by suppressed immune cell function, dense collagen deposition, and alterations in mitochondrial metabolism.

**Conclusions:**

ecPICK provides a scalable, budget-conscious platform for ecDNA mapping without the need for high-cost sequencing. By revealing the spatial remodeling of the tumor landscape, it represents a powerful tool for rapid patient stratification and novel insights into ecDNA-mediated malignant progression.

## Introduction

Extrachromosomal DNA (ecDNA) is a circular DNA molecule that exists outside of the chromosomes. It is involved in oncogene amplification 1, carcinogenesis 2, progression 3, treatment resistance 4, 5, and intratumoral heterogeneity 6 and is frequently observed in many cancers 7, 8. Numerous studies have addressed the significance of ecDNA in cancer 9, 10. Nevertheless, it is difficult to detect ecDNA in clinical tissue samples.

Whole-genome sequencing (WGS) and fluorescence in situ hybridization (FISH) are two methods to detect ecDNA in tissues; however, neither of these methods is ideal for clinical use. Although WGS can measure the overall ecDNA content, it cannot identify the structural characteristics of ecDNA within individual cells 11. In addition, the high cost, long turnaround time, and complicated analytical processes prevent its widespread use. While FISH is capable of achieving a subcellular resolution of approximately 200 nm 12, it is inherently limited by its dependence on specific DNA sequences for detection. Thus, each FISH assay can typically target only a single, known ecDNA species and is unable to detect all ecDNAs, particularly those with unknown sequences. Because of these methodological constraints, only a limited number of cancer centers have the capability for systematic ecDNA testing. Therefore, integrating ecDNA detection into routine pathological workflows is difficult, severely hindering both clinical translation and fundamental research in the ecDNA field. Bridging this technological gap requires innovative approaches.

Because of the complexity and high heterogeneity of ecDNA, its comprehensive detection by traditional morphological analysis alone is challenging. In recent years, artificial intelligence-based image analysis methods have advanced rapidly for clinical diagnostics 13-17. Based on these developments, we developed ecPICK (Pathological Insight from Cytomorphology and Karyotype), a deep learning-based spatial diagnostic platform designed to predict ecDNA status directly from H&E-stained, whole-slide images (WSIs). Using a two-stage architecture integrating ResNet and DNN 18 to decipher complex cytomorphological features, ecPICK provides a clinically actionable output, which includes a whole-slide ecDNA probability score for rapid patient stratification and spatial distribution heatmap to visualize the ecDNA landscape within the tumor microenvironment. This approach transforms standard pathology slides into multidimensional diagnostic assets, effectively bridging the gap between molecular characteristics and routine clinical practice. Technically, ecPICK achieves a triple paradigm shift. First, it requires only standard H&E-stained slides as the input, which significantly lowers the technical barrier, and second, it reduces the analysis time to approximately 2–3 min per sample, which markedly improves efficiency compared with WGS. Finally, it substantially reduces the cost per sample. Validated on a pan-cancer cohort of 4,280 H&E-stained WSIs across 20 cancer types, the model demonstrated excellent generalizability and maintained robust performance in an independent intrahepatic cholangiocarcinoma (ICC) cohort. The establishment of this “morphology–molecular” correlation opens new dimensions for studies on the tumor microenvironment.

## Methods

### ecPICK architecture

#### (1) Model Design

The ecPICK framework is engineered around a dual-stage ResNet-DNN architecture optimized for histopathological image analysis. This pipeline integrates a ResNet backbone 19 acting as a high-fidelity feature extractor to distill complex semantic patterns from medical imagery, which are subsequently processed by a deep neural network (DNN) head for quantitative classification.

ResNet Feature Extraction Backbone: To circumvent the vanishing gradient challenges inherent in deep convolutional networks, we utilized a ResNet architecture characterized by residual skip connections. This design facilitates the stable training of deep hierarchical representations by allowing direct gradient flow across layers. The extractor performs multi-scale convolutional operations, systematically transforming raw input pixels into a rich feature space—transitioning from granular edge textures to high-level semantic abstractions through successive layers of hierarchical learning.

At the end of the ResNet pipeline, we used a global average pooling (GAP) operation to turn two-dimensional spatial maps into a flattened, one-dimensional feature representation. The GAP method is very different from regular fully connected layers because it cuts down on the number of parameters while still keeping important spatial invariance, which helps prevent model overfitting. This pooling mechanism turns each input image into a 2048-dimensional latent vector that effectively captures the core semantic descriptors of the histopathological landscape.

For this analysis, we used the ResNet-50 architecture with pre-trained weights to quickly extract features from standard H&E-stained WSIs. To make the model more sensitive to tissue-specific patterns, we used a stratified fine-tuning strategy to bridge the gap between natural images and the specialized histopathological context. This transfer learning approach made sure that the pre-trained parameters were gradually adjusted to the subtle textural and morphological signatures found in medical imaging.

We designed a dedicated multilayer perceptron (MLP) to serve as the classification head following the feature extraction module. This network receives the 2048-dimensional latent embeddings from the ResNet backbone and processes them through 2–3 sequential functional modules. Each module is structurally identical, comprising: (1) a batch normalization layer to stabilize covariate shift, (2) a fully connected layer with 2048 neurons to preserve feature dimensionality, and (3) a ReLU activation function to introduce non-linearity. This hierarchical architecture, totaling approximately 8.4 million trainable parameters, effectively decodes the complex morphological signatures of ecDNA from the high-dimensional feature space into the final predictive output.

The architecture incorporates a modular design framework 20-22, wherein each functional unit is composed of a batch normalization layer, a fully connected layer, and a non-linear activation function. Within these modules, the input features undergo initial batch normalization to stabilize internal covariate shift and ensure training consistency. This is succeeded by a linear transformation through the fully connected layer, which is configured with 2048 neurons to maintain the feature dimensionality of the input vector. Subsequently, a secondary batch normalization operation is applied to the transformed signal, culminating in a non-linear mapping facilitated by the activation function to enhance the model's representational capacity.

The strategic implementation of batch normalization throughout the architecture serves a dual purpose: it accelerates the convergence of stochastic gradient descent while acting as an implicit regularizer to enhance the model's generalizability across various clinical datasets. The DNN classification head contains around 8.4 million trainable parameters, most of which are found in the dense weight matrices of the fully connected layers. These parameters give the network the representational depth it needs to decode and parameterize the complex feature signatures found in ecDNA-driven pathologies. It is explicitly noted that this classification head operates on tile-level independent predictions without a 512-dimensional hidden layer.

#### (2) Model Training

To improve the transfer learning process 23-25, we used a two-phase fine-tuning protocol that was stratified. In the first phase, we froze the entire ResNet backbone to keep the pre-trained feature hierarchies and focused all computational resources on optimizing the weights of the new DNN classifier. This isolated training phase made sure that the classification head could effectively map fixed ImageNet-derived embeddings to histopathological categories. When performance reached a plateau, we moved on to the second phase, where we unfroze the last two convolutional blocks of the ResNet. This targeted relaxation allowed the high-level feature extractors to undergo domain-specific recalibration, fine-tuning the model's sensitivity to the unique textural nuances of medical imagery. To keep training balanced, we used a decoupled learning rate schedule 26-28: a higher magnitude was given to the DNN for quick initialization, while a much lower rate was given to the unfrozen convolutional layers to prevent catastrophic forgetting of the pre-trained weights. The system was optimized end-to-end, using early stopping and weight decay to avoid overfitting and ensure strong performance across independent cohorts.

#### (3) Model Interpretation

To make the model easier to understand, we used the SHapley Additive exPlanations (SHAP) framework, which is based on cooperative game theory 29, to look at the 2048-dimensional feature embeddings that the ResNet backbone created. This method allows for a thorough attribution analysis, measuring how much each individual feature dimension contributes to the final predictive logit. By back-projecting these importance scores from the latent feature space onto the original pixel domain, we created spatially-resolved interpretability heatmaps. This visualization pipeline effectively decodes the model's "decision-making" process, pinpointing the specific pathological topographies and cytomorphological signatures within the H&E-stained WSIs that have the most determinative influence on ecDNA status prediction. To bridge the gap between high-dimensional features and observable pathological outcomes, a systematic mapping and screening workflow was established. First, the 2048-dimensional mathematical weights from the SHAP interpreter were projectively mapped back onto the original native coordinate space of the digital slide to identify local hot spots. Subsequently, a double-blinded visual inspection under high-power magnification was conducted across the cohort to screen and categorize the recurrent, predominant cytomorphological alterations unique to these high-attribution zones. These categorizations were further verified through spatial co-localization with independent interphase FISH experiments.

To spatially localize the model's focus, we utilized Gradient-weighted Class Activation Mapping (Grad-CAM) 30, which determines the most significant image regions by utilizing the gradient flow from the classification layer. By computing the gradients of the target category score relative to the final convolutional feature maps, the algorithm produces a class activation heatmap. This visualization effectively emphasizes the prominent morphological domains prioritized by the network, providing an intuitive representation of the "attentional focus" associated with each diagnostic prediction.

In our configuration, the final convolutional layer of the ResNet backbone was designated as the target for gradient-weighted feature extraction. We computed the importance weight for each feature channel by applying global average pooling (GAP) to the back-propagated gradients of the target class with respect to that layer’s output. These scalar weights were then integrated with the forward-pass feature maps via a weighted linear combination to synthesize a coarse localization map. To isolate features with a positive contribution to the target class, a ReLU (Rectified Linear Unit) activation was applied to suppress negative intensity values. Finally, the resulting map underwent upsampling—typically through bilinear interpolation—to the original input tile dimensions, yielding a continuous spatial importance score for each pixel without any auxiliary indexing, enabling precise visual correlation between morphological features and ecDNA predictions.

The Grad-CAM heatmap is combined with the original H&E-stained image using a pseudo-color overlay to show spatial attribution. In this representation, the color spectrum acts as a gradient of diagnostic significance: "hot" red areas show the morphological domains that had the biggest effect on the model's output, while "cool" blue areas show the areas that had the least effect on the model's output. This chromatic mapping allows for a direct, high-contrast correlation between local tissue architecture and the network's automated decision logic.

### H&E-stained image processing

To safeguard the model's performance against technical heterogeneity, all WSI-derived tiles were processed using Macenko color normalization, effectively standardizing the idiosyncratic H&E staining profiles inherent to different clinical centers and digital scanners. This pre-processing was augmented by a rigorous training-time regularization regime; specifically, we implemented a multifaceted stochastic augmentation pipeline encompassing random axial flips (horizontal and vertical), arbitrary rotations, and color jittering—the latter parameterized by a 0.25 variance factor across brightness, contrast, and saturation. These interventions were strategically deployed to suppress latent batch effects and ensure that the network's latent space converges on conserved cytomorphological hallmarks of ecDNA rather than spurious technical artifacts.

This study utilized conventional H&E-stained histopathological sections as the primary data source. The computational preprocessing workflow began with color normalization to address inconsistencies in staining between batches. This was followed by Region of Interest (ROI) segmentation to separate malignant areas. We used a multi-scale, tiered tile extraction strategy to capture a range of visual descriptors, from fine edge textures to high-level semantic abstractions. The combined dataset was split into training and validation subsets using a decoupled 8: 2 ratio. The specific operational framework is detailed below:

(1) Data Preparation and Quality Control: SVS format images with a resolution of 0.5 µm/pixel (20× magnification) were prioritized. Slides with invalid regions were excluded, and only those with a tissue area coverage ≥ 80% were retained; otherwise, they underwent manual review.

(2) Color Calibration: One to two slides exhibiting "average staining" were selected as templates. The Macenko method was applied in the LAB color space to compute inter-batch staining variability.

(3) Patching and Annotation Alignment: A sliding window approach was used to generate tiles of 512 × 512 pixels with a stride of 256 pixels (50% overlap). Only the central 256 × 256 pixel region of each tile was saved to minimize boundary artifacts. Background tiles were filtered out.

(4) Data-Level Augmentation for Training Set: Augmentation techniques included random rotations (90°/ 180°/ 270°), horizontal/vertical flipping; HSV adjustments (hue ± 6, saturation scaling between 0.8-1.2) to simulate staining intensity variations; and random elastic deformation (grid = 3, sigma = 15) to enhance robustness to deformations.

(5) Validation: The dataset partitioning into training and validation sets was implemented using strict patient-level stratification. In practice, the split was performed at the WSI level, where each WSI strictly corresponds to a patient in the TCGA pan-cancer cohort. This protocol explicitly prevents any patch-level data leakage, ensuring that all downsampled images from a single individual are allocated exclusively to either the training or testing framework. To make the model more robust against technical changes (batch effects), the patches were processed with a lot of data augmentation, such as random rotations, flips, and color jittering (changing brightness and contrast within a range of ± 0.2). A visual inspection of 100 augmented patches was done to make sure nuclear clarity was kept. The distribution of patch-level labels was statistically analyzed, and weighted sampling or dynamic sampling 31, 32 was used if necessary to fix class imbalance.

### Model evaluation and validation

We used a stratified five-fold cross-validation protocol on the TCGA discovery cohort (n = 4,280) to make sure that the model evaluation was strong and the performance estimates were accurate. We used the Area Under the Receiver Operating Characteristic Curve (AUROC), F1-score, Precision, Recall, and Specificity to fully benchmark the model's performance. To check the statistical stability of these estimates, we used bootstrap resampling (1,000 iterations) to get 95% confidence intervals. In addition to internal cross-validation, we rigorously tested the model's trans-institutional generalizability through external validated on an independent cohort of 134 ICC patients (268 WSIs) from Tianjin Cancer Hospital.

### Quantification and statistical analysis

We used R (version 4.3.0) and GraphPad Prism (version 9.0) to do statistical calculations and make graphs. We reported continuous metrics as either medians with interquartile ranges (IQR) or means ± standard deviation (SD), depending on what was appropriate. For comparative analyses of continuous data, we used Student's t-tests for parametric distributions and Mann-Whitney U tests for non-parametric datasets. We used Chi-square tests or Fisher's exact tests, depending on cell frequencies, to look for differences in categorical variables.

To assess clinical outcomes, we generated Kaplan-Meier survival curves, with statistical significance adjudicated via the log-rank test. To identify independent prognostic factors, Cox proportional hazards models were constructed, adjusting for clinically relevant covariates, including tumor grade and TNM stage. Statistical significance was defined by a two-sided *P* < 0.05. To minimize the risk of Type I errors in high-dimensional analyses, *P*-values were adjusted using the Benjamini-Hochberg false discovery rate (FDR) procedure. All underlying model assumptions, including proportionality and normality, were rigorously validated through residual diagnostics and Q-Q plots.

### Ethics oversight

The study was approved by the Ethics Committee of Nankai University, approval number: NKUIRB2025058. Informed consent for this project was waived by the respective ethics commissions because this study only involves a retrospective anonymized analysis of archival pathology samples. The tissue microarrays utilized in this study were approved by the Ethics Committee of Changsha Yaxiang Biotechnology Co., Ltd. (Ethics Approval No.: Csyayj2024071).

## Results

### Overview of ecPICK

**Figure 1:**
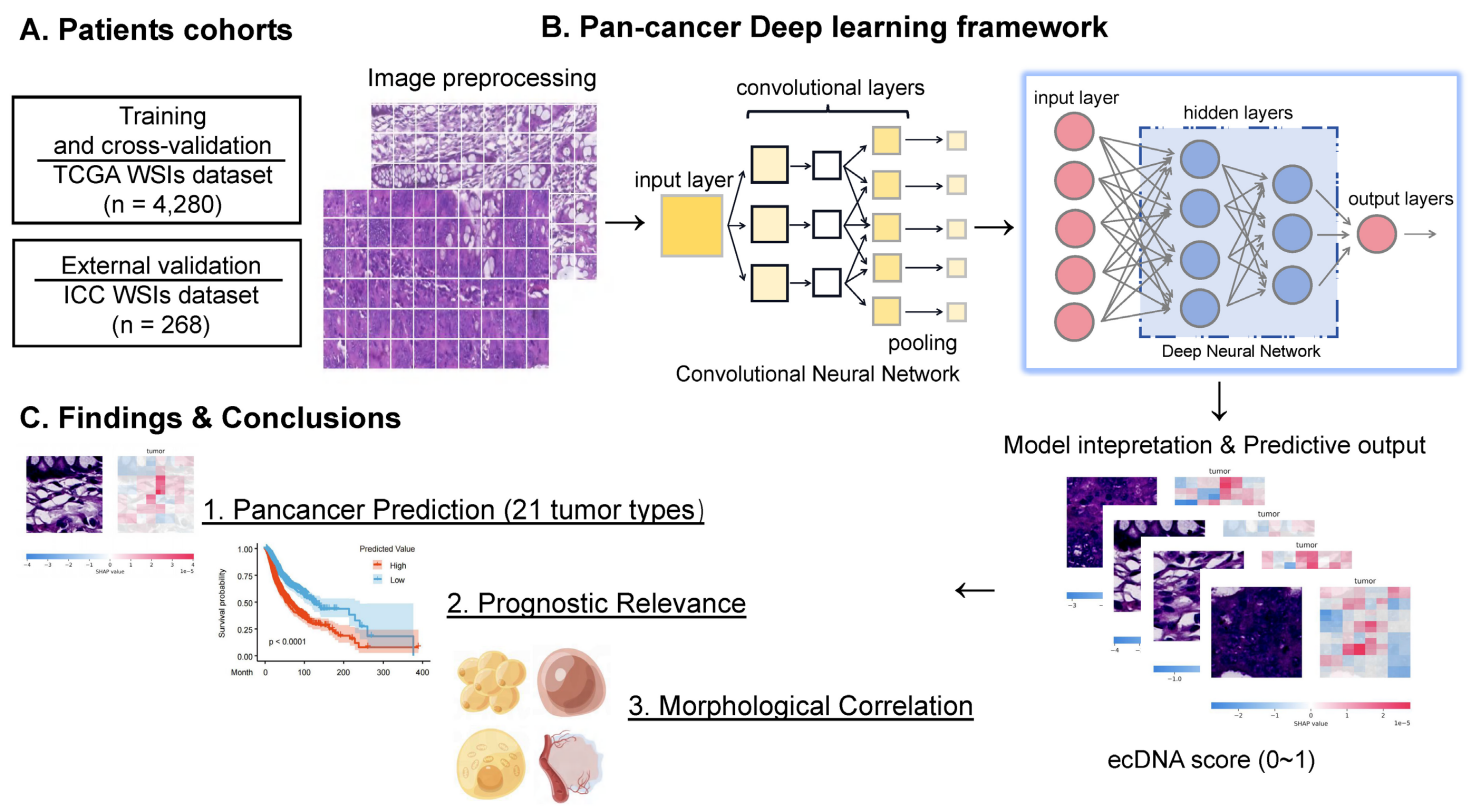
** ecPICK model architecture, training data, and interpretability analysis. (A)** Study cohorts and data partitioning. The model was trained and tested on 4,280 H&E WSIs from the TCGA pan-cancer cohort (20 cancer types), split 8: 2 into training and test sets. External validation used 268 intrahepatic cholangiocarcinoma WSIs from Tianjin Cancer Hospital. **(B)** Algorithmic workflow and technical details. (i) Whole-slide preprocessing: adaptive background detection (Otsu thresholding) and Macenko color normalization were applied; tissue was segmented into 512 × 512 pixels, achieving > 99.5% tissue retention. (ii) Each 512×512 tile is independently processed: ResNet-50 extracts a 2048-dim feature vector via per-tile global average pooling (GAP), which is then fed into the DNN classifier to yield tile-level ecDNA probabilities. (iii) Interpretability: SHAP identified significant predictive regions and generated high-resolution feature attribution heatmaps. The ecDNA prediction probability output is quantified as a score (range: 0-1). **(C)** Output visualization and quantitative analysis. Cellular-level SHAP heatmaps display the spatial distribution of ecDNA prediction probabilities and morphological feature attributions in representative H&E tissue regions. The model predicted 21 cancer types, including 20 data sources derived from TCGA and 1 independent cohort dataset. Kaplan–Meier survival analysis and Cox regression confirmed a significant association (*P* < 0.05) between high ecDNA prediction scores and poor clinical prognosis. A quantitative model linking cytomorphological features to ecDNA content was established to assess tumor heterogeneity and malignant progression.

To overcome the technical limitations of conventional ecDNA detection, we proposed ecPICK, a deep learning framework based on a convolutional neural network, designed to predict ecDNA abundance from routine H&E-stained WSIs. The model was trained using the WSI dataset from the Cancer Genome Atlas, which encompasses 20 cancer types with a total of 4,280 training and testing images (training-to-testing ratio: 80%: 20%). The ecDNA-negative and positive labels used in the TCGA data by ecPICK originate from Hoon Kim's study 7. External validation was done with an independent cohort of 268 WSI of intrahepatic cholangiocarcinoma provided by the Tianjin Cancer Hospital (Figure 1A). The algorithmic workflow of ecPICK is based on a two-stage design (feature extraction and classification prediction). Its computational framework is detailed as follows (Figure 1B):

(1) Whole-Slide Preprocessing and Tile Segmentation: Input WSIs were subject to standard preprocessing. An adaptive background detection algorithm 33 was used to remove blank areas (based on Otsu threshold segmentation). Color normalization was done using Macenko's method to eliminate staining variations. Tissue tiles were segmented into 512 × 512 pixels. This pipeline ensures a tissue retention rate of > 99.5%, which significantly enhances the robustness and consistency of feature extraction.

(2) Multi-Scale Morphological Feature Extraction and Classification Prediction: For the feature encoding stage, a pretrained ResNet-50 was used as the backbone network, with its original fully connected layers removed. The hierarchical convolutional modules progressively extracted multilevel morphological information, ranging from low-level edge textures to high-level semantic features. Global average pooling was applied at the end of ResNet, compressing each image tile into a 2,048-dimensional feature vector, thereby preserving key spatial information while reducing the number of parameters. For the classification prediction stage, a deep fully connected neural network (DNN) was designed as the classifier. Taking the 2,048-dimensional feature vector from ResNet as input, the network contained multiple processing modules. Each module consisted of a batch normalization layer, a fully connected layer (2,048 neurons), and an activation function. Non-linear transformations within these modules yielded tile-level ecDNA presence probabilities. The entire DNN classifier comprised approximately 8.4 million trainable parameters and employed a layered fine-tuning strategy via transfer learning to adapt to medical image features. The final output was mapped to the [0, 1] interval through a Sigmoid function, representing the ecDNA probability.

(3) Training Optimization and Interpretability Analysis: A patient-level stratified five-fold cross-validation protocol was utilized to partition the master dataset into independent training (80%) and testing (20%) cohorts, ensuring robust evaluation. The Adam optimizer (learning rate 1e-4) was used to optimize the model, and binary cross-entropy was used as the loss function. To determine how the model made decisions, gradient-weighted Class Activation Mapping (Grad-CAM) technology was used to identify areas with a big impact on predictions. Based on this, visual heatmaps were constructed to make the model easier to understand and more useful in the clinic.

Through an in-depth analysis of the model outputs, we determined the key value of ecPICK in ecDNA spatial localization, prognostic prediction, and association with cellular morphology (Figure 1C). First, the Shapley Additive exPlanations (SHAP) algorithm was used for attribution analysis of the model predictions, generating ecDNA spatial localization heatmaps for H&E-stained slides. Next, profound analysis revealed that ecPICK-based predictions contain significant independent prognostic value. Its predictive capability was consistent with established clinical standards, such as AJCC cancer staging and histological grade, which indicates that ecDNA serves as a novel, noninvasive prognostic biomarker with substantial potential for clinical application. Furthermore, this study is the first to identify a close association between the presence and content of ecDNA and specific cellular morphological features. This enables the indirect analysis of ecDNA distribution and enrichment from a cytomorphological perspective, circumventing the limitations of traditional molecular biology techniques.

### Performance of ecPICK in the TCGA validation set

**Figure 2:**
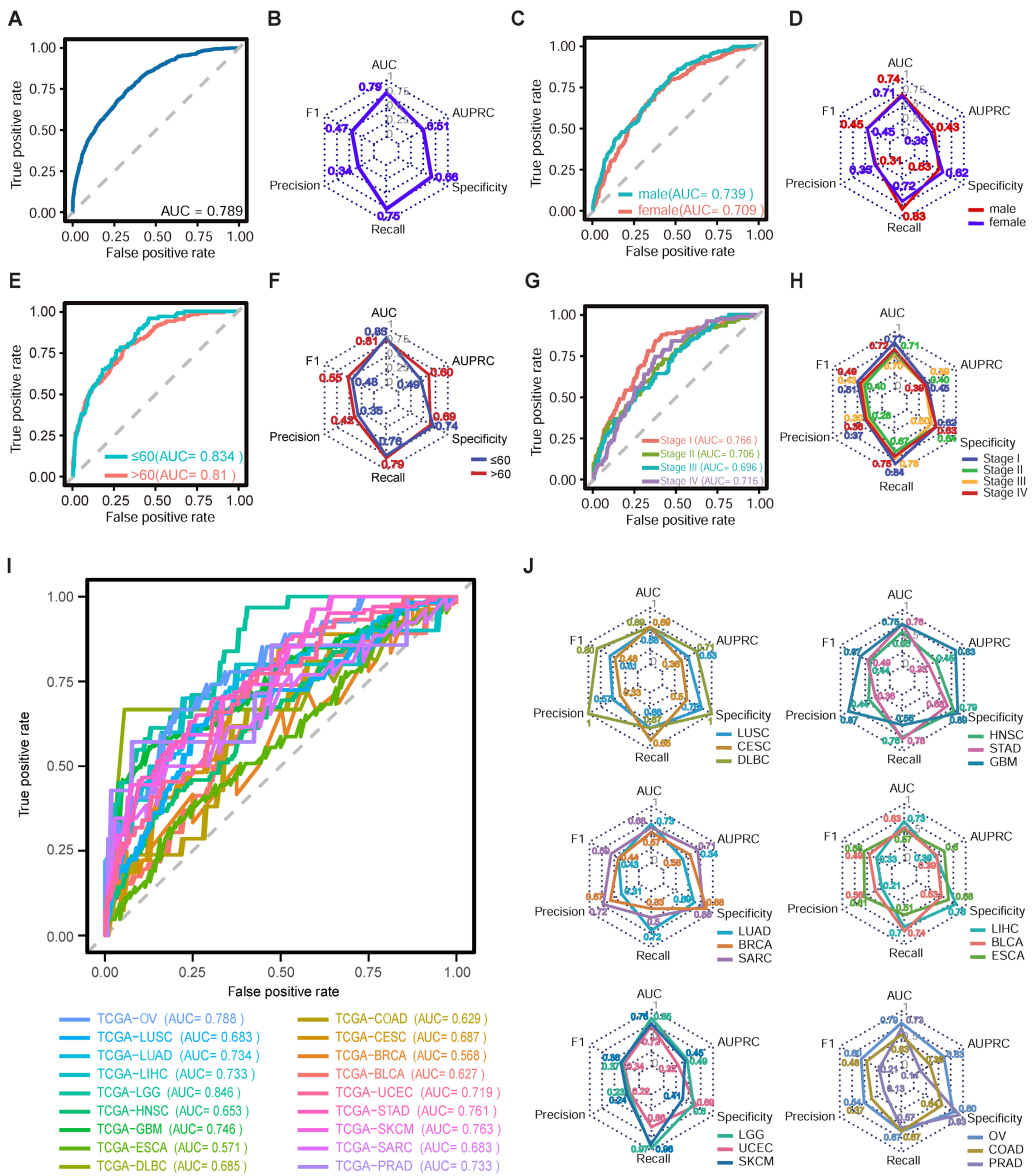
** Performance evaluation of ecPICK across multiple dimensions in the TCGA cohort. (A)** ROC curve for overall ecPICK performance across 20 cancer types (n = 4,280; TCGA); AUC = 0.789 (95% CI: 0.773–0.805). **(B)** Radar plot of six core metrics for the full cohort: AUC (0.79), AUPRC (0.51), F1-score (0.47), Precision (0.34), Recall (0.75), Specificity (0.66); axes normalized to 0–1.** (C)** ROC curves by sex: male (n = 2,266, AUC = 0.739; 95% CI: 0.710–0.768) and female (n = 2,014, AUC = 0.709; 95% CI: 0.678–0.740). **(D)** Radar plot comparing six metrics by sex; the male group showed higher AUPRC (0.43) and Recall (0.83); the female group showed higher Precision (0.33) and Specificity (0.62). **(E)** ROC curves by age: ≤ 60 years (n = 1,258, AUC = 0.834; 95% CI: 0.810–0.868) and > 60 years (n = 1,516, AUC = 0.810; 95% CI: 0.778–0.840). **(F)** Radar plot by age; the > 60 years group outperformed in AUPRC (0.60), Recall (0.79), F1-score (0.55), and Precision (0.42). **(G)** ROC curves by AJCC stage: I (AUC = 0.766), II (AUC = 0.706), III (AUC = 0.696), IV (AUC = 0.716).** (H)** Radar plot by AJCC stage; Stage I samples performed best overall. **(I)** Individual ROC curves for each cancer type; the best-performing models were LGG (AUC = 0.85), SKCM (AUC = 0.76), and STAD (AUC = 0.76). ROC curves could not be plotted for KIRC and THCA due to the absence of ecDNA-positive patients; however, these datasets were still used for training ecPICK with TCGA data. **(J)** Radar plots of model performance by cancer type; GBM: best AUPRC (0.83); DLBC: highest F1-score (0.80), Precision (1.00), Specificity (1.00); LGG: highest Recall (0.968).

The performance of ecPICK was evaluated by plotting the receiver operating characteristic (ROC) curve. The model was trained with 4,280 H&E WSIs encompassing 20 tumor types from the TCGA dataset. The assessment based on five-fold cross-validation revealed that ecPICK achieved a macro-average area under the ROC curve (AUROC) of 0.789 (95% CI: 0.773–0.805; Figure 2A), indicating its robust capability in discriminating ecDNA-positive from ecDNA-negative samples. For a more comprehensive evaluation, we systematically assessed multiple key metrics, including the F1 score, precision, recall, specificity, and the area under the precision-recall curve (AUPRC) (Figure 2B). Consistent results across these metrics indicate that ecPICK maintains high sensitivity and good specificity.

To evaluate the model’s generalizability, we performed a multi-dimensional subgroup analysis using the TCGA dataset, categorized by sex (male: 2,266; female: 2,014), age (≤ 60 years: 1,258; > 60 years: 1,516), AJCC cancer stage (Stage I: 1,157; Stage II: 731; Stage III: 908; Stage IV: 1,484), and 20 distinct tumor types. ROC curves were generated, and key metrics were evaluated for each subgroup (Figure 2C–J). The results indicated a balanced performance across sex and age subgroups (male AUROC = 0.739, female AUROC = 0.709, Figure 2C; ≤ 60 years AUROC = 0.834, > 60 years AUROC = 0.81, Figure 2E). The ecPICK tool was able to detect ecDNA at all stages of cancer [mean AUC 0.70 ± 0.03 (95% CI: 0.68-0.72)], but it was better at predicting early-stage tumors (Stage I–II AUROC = 0.741) compared with late-stage tumors (Stage III–IV AUROC = 0.729; Figure 2G). This is consistent with the biological characteristics of ecDNA during tumor evolution, in which early-stage ecDNA shows high clonality, structural homogeneity, and distinct morphological signals. The analysis of 20 different tumor types revealed that ecPICK predicted ecDNA well for various cancers (Figure 2I). The model showed excellent generalizability and robustness by correctly predicting cancers. It was particularly good at predicting low-grade glioma (LGG), skin cutaneous melanoma (SKCM), and stomach adenocarcinoma (STAD), with AUROCs of 0.85, 0.76, and 0.76, respectively. To confirm the model’s stability, it was tested using multiple metrics, such as the F1-score and precision, based on the data that corresponded to the ROC curves for different subgroups (Figure 2D, F, H, J). The results indicated that ecPICK consistently showed good performance across all subgroups, which further supports its potential as a tool for ecDNA detection in different clinical situations.

To thoroughly assess the generalizability and robustness of ecPICK, we created an independent external dataset that was validated on an independent cohort of 134 patients with ICC (268 WSIs). Although the TCGA study included specimens of intrahepatic cholangiocarcinoma, the ecDNA status was not determined. Consequently, these samples were excluded from the training set of the present study and were allocated under another independent validation cohort. Because Hoon Kim’s study 7 did not include ecDNA analysis on the TCGA-CHOL cohort, these samples were excluded from the training process of this model. The current cohort consisted of formalin-fixed paraffin-embedded (FFPE) tissue samples obtained from patients with ICC who underwent surgical resection at Tianjin Medical University Cancer Institute and Hospital. The model achieved an outstanding macro-average AUROC of 0.913 (95% CI: 0.898–0.927) in distinguishing ecDNA-positive from ecDNA-negative ICC samples (Figure S1A). A multi-dimensional performance evaluation revealed that ecPICK achieved perfect recall (1.0) while maintaining high specificity (0.91), with an F1-score of 0.67 and precision of 0.50 (Figure S1B). These results demonstrate that the model exhibits predictive accuracy, reliability, and robustness in real-world clinical environments. A violin plot (Figure S1C) illustrates the distribution disparity of the model’s prediction scores between ecDNA-positive and negative cohorts, with the median score significantly elevated in the positive group, which further substantiates the discriminative efficacy of ecPICK for ecDNA status. To convert the AUROC (0.789) into practical clinical insights, we assessed ecPICK using a three-tier stratification framework (Figure S1D–F). By establishing dual high-confidence thresholds, a rule-out bound at 0.143 (high sensitivity) and a rule-in bound at 0.258 (high specificity), we effectively categorized the pan-cancer cohort into three risk levels. Notably, ecPICK acted as an efficient “digital filter,” enabling the immediate exclusion of 31.8% of clear negative cases while maintaining a high safety margin. Approximately 17.6% of the samples were identified as high-risk, whereas the remaining 50.6% were considered a “Gray Zone” for WGS or FISH validation (Figure S1F).

### Association of ecPICK prediction scores with clinical prognosis

Because ecDNA is associated with reduced survival in patients with various cancers, we determined the clinical relevance of ecPICK using an independent clinical cohort. We collected H&E-stained WSIs from 75 patients with hepatocellular carcinoma and 80 patients with colorectal cancer (CRC; two slices from each patient) at the Tianjin Medical University Cancer Hospital and the First Affiliated Hospital of Nankai University. Each sample was analyzed using ecPICK to obtain the probability of ecDNA presence (i.e., model-predicted ecDNA score). Next, the correlation between the ecPICK-predicted ecDNA score and patient prognosis, tumor stage, and treatment response were determined through statistical analysis.

**Figure 3:**
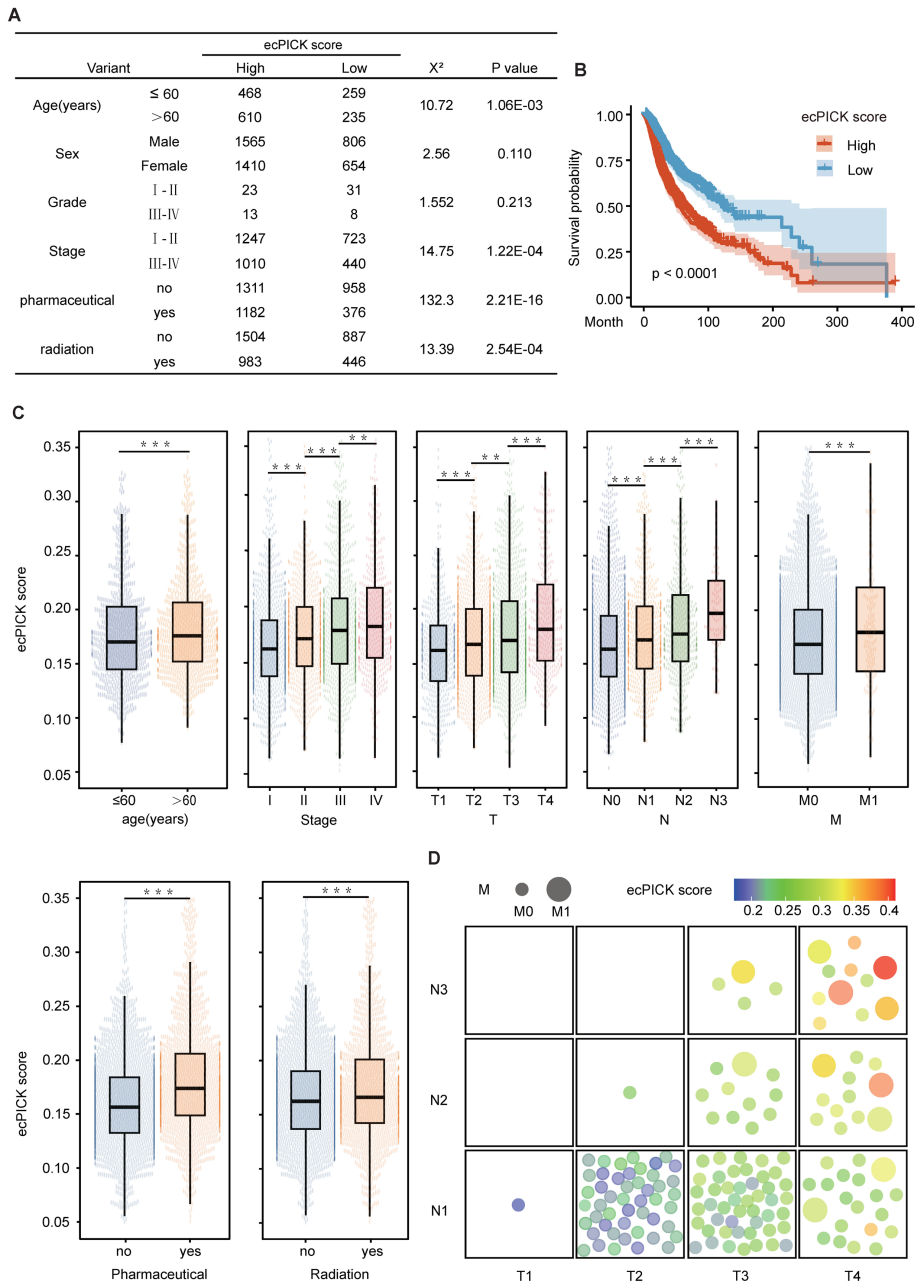
** Integration of multi-cohort H&E whole-slide image data and association analysis between ecPICK score and clinical characteristics, survival outcomes, and TNM staging. (A)** Clinical feature table from the TCGA pan-cancer cohort (n = 4,280), HCC (n = 150), and CRC (n = 160). The ecDNA score differed significantly across age strata (χ² = 10.72), AJCC stage (χ² = 14.75), radiotherapy (χ² = 132.3), and chemotherapy (χ² = 13.39); all *P* < 0.001. **(B)** Kaplan–Meier survival curves stratified by median ecPICK score; the high-score group had significantly shorter median survival (log-rank *P* < 0.001). **(C)** Multi-factor stratified box plots showing positive correlations between ecPICK score and AJCC stage, TNM grade, and treatment status; patients > 60 years showed higher ecDNA content than younger individuals. **(D)** Bubble plot of TNM staging in 155 HCC/CRC patients (each patient has two slices): x-axis = increasing T stage; y-axis = increasing N stage; bubble size indicates M stage (small = M0, large = M1); color gradient reflects ecDNA scores (blue = low, red = high). High-score/late-stage patients cluster upper-right; low-score/early-stage patients cluster lower-left.

This cohort included a sufficient number of patients with different age distributions and histological grades, ensuring an adequate sample size for robust statistical analysis within the strata of various clinicopathological characteristics. Stratified analyses for individual factors were conducted to categorize patients by age (≤ 60 vs. > 60 years), sex, histological grade, clinical stage (Stage I–II vs. III–IV), and treatment status (received chemotherapy/radiotherapy vs. did not receive). Because of missing data in some grouped datasets within the open-source data, the sum of the total group counts was less than the total measurement quantity. Using the median prediction score as a cutoff, the patients were divided into high and low prediction score groups, and a chi-square test was used to analyze the distribution differences of the clinical characteristics between the groups (Figure 3A). The results indicated that older patients (> 60 years) had significantly higher ecDNA scores (*P* < 0.001), and individuals with advanced-stage (Stage III–IV) disease exhibited markedly higher ecDNA scores compared with early-stage patients (*P* < 0.0001). Those undergoing radiotherapy/chemotherapy had considerably increased ecDNA scores compared with those who did not receive treatment (*P* < 0.001). Kaplan–Meier survival analysis (Figure 3B) revealed that patients with high ecDNA scores had a significantly reduced overall survival rate (log-rank *P* < 0.0001).

The box plot in Figure 3C illustrates the distribution patterns of the scores by integrating key clinical variables, including a stepwise increase from Stage I to Stage III (Kruskal–Wallis *P* < 0.0001), with significantly elevated scores associated with advanced TNM stage (T1→T4, N0→N2, M0→M1), and markedly higher scores in the group that underwent chemotherapy/radiotherapy compared with the group that did not (Mann–Whitney *P* < 0.001).

The relationship between ecDNA scores and TNM stage was visualized using a bubble plot (Figure 3D). Increases in T, N, and M stage dimensions were positively correlated with ecDNA score. These results indicate that the ecPICK-predicted ecDNA score is strongly associated with clinical stage, treatment response, and survival prognosis. Furthermore, multivariate survival analysis suggests that the ecDNA score is an independent prognostic marker for malignant tumors.

### ecPICK prediction score holds independent clinical prognostic value

**Figure 4:**
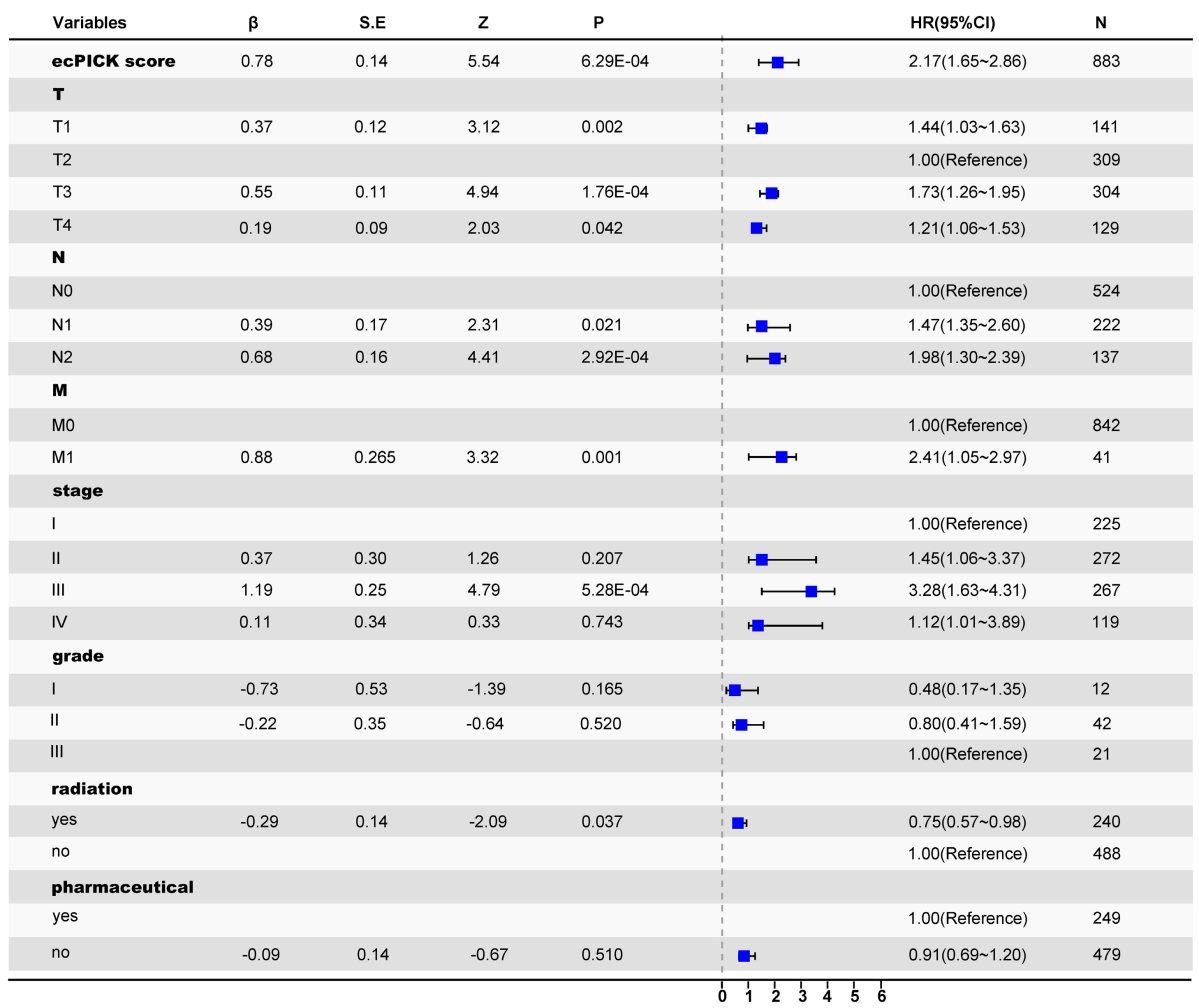
** Cox proportional hazards regression forest plot.** Forest plot with hazard ratio (HR) on the horizontal axis and 95% confidence intervals (CI) indicated by line segments. The model prediction score was treated as a continuous exposure variable. An increase in score was significantly associated with elevated mortality risk (β = 0.78, HR = 2.17, 95% CI: 1.65–2.86); each one-unit score increases corresponded to a 117% increase in mortality risk.

After confirming the strong correlation with various tumor prognostic characteristics, the independent prognostic significance of ecPICK was assessed via a multivariable framework. Multivariable Cox proportional hazards regression analysis with mitigated overfitting indicated that the ecPICK prediction score was a significant independent prognostic factor (Figure 4). In particular, the ecPICK prediction score corresponded to a regression coefficient (β) of 0.78 (standard error S.E. = 0.14, Wald test Z = 5.54, *P* < 0.001), with a hazard ratio (HR) of 2.17 (95% CI: 1.65–2.86). This indicates that after adjusting for other clinicopathological variables, such as tumor grade and TNM stage, each one-unit increase in the ecPICK prediction score was associated with an approximate 2.17-fold increase in risk of death, suggesting its strong capability of risk prediction.

Within the multivariable model that included established gold-standard prognostic indicators, such as tumor grade and TNM stage, the ecPICK prediction score exhibited significant prognostic discriminatory power. The confidence interval for its hazard ratio did not include 1, and its predictive performance was comparable to, if not superior to, that of the traditional indicators. These results indicate that the ecPICK-derived ecDNA load prediction score may serve as an independent prognostic biomarker for clinical translation.

### Characterization of ecDNA levels through cancer cell morphology

**Figure 5:**
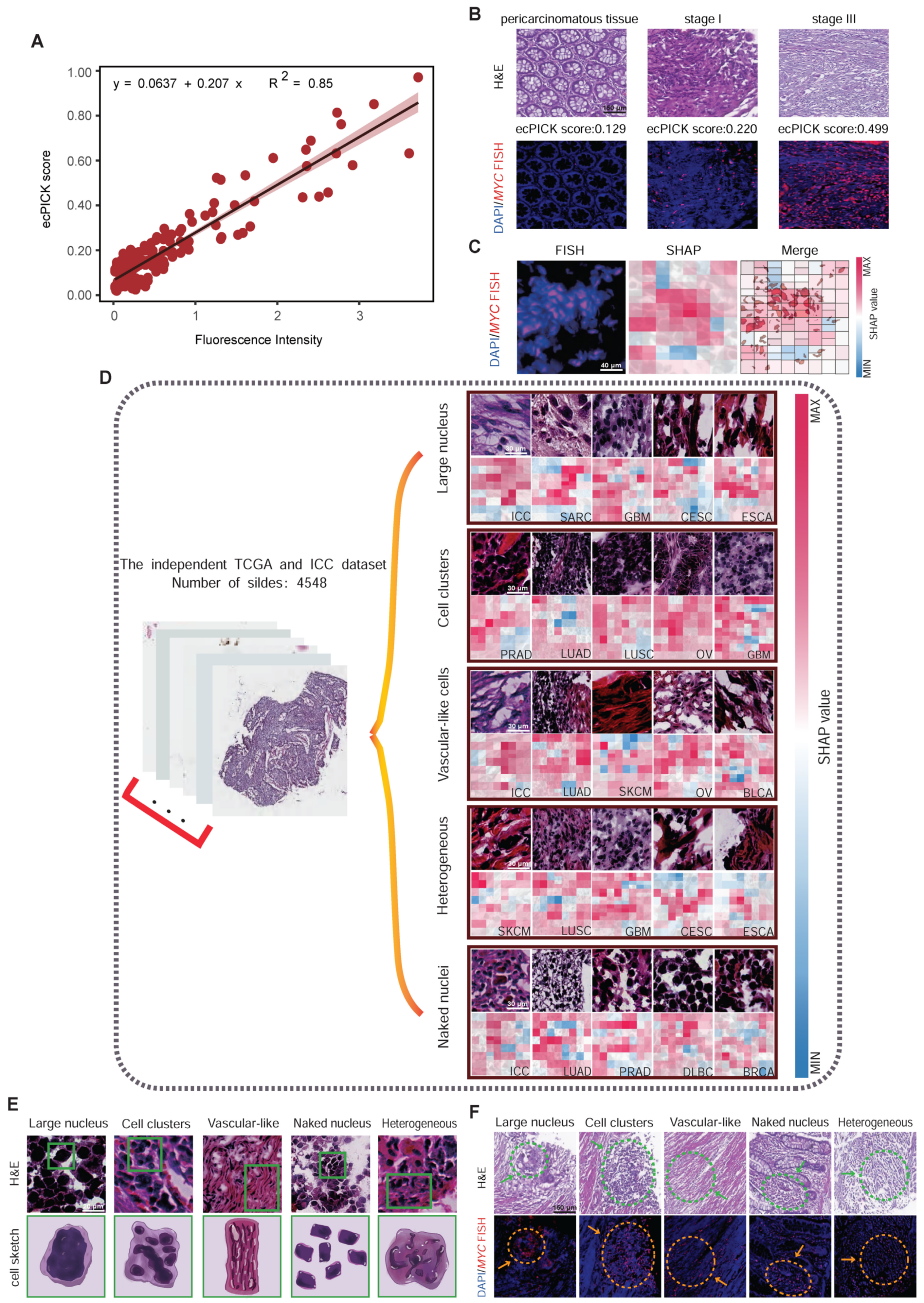
** Association between ecDNA and Cellular Morphology. (A)** Scatter plot showing a significant positive linear correlation (R² = 0.85, *P* < 0.0001) between ecPICK prediction score and MYC fluorescence intensity by FISH in a 160-core CRC tissue microarray (80 paired CRC and adjacent normal tissue samples); regression slope β = 0.207.** (B)** Comparative visualization of ecPICK score gradient (low to high) aligned with corresponding MYC FISH fluorescence intensity; a 0.1-unit increase in ecPICK score associated with a 38% average increase in fluorescence intensity (95% CI: 32–44%). **(C)** Co-localization analysis between MYC FISH expression and ecPICK SHAP heatmaps; regions with high MYC expression (fluorescence intensity > 8) showed significantly higher SHAP values (0.32 ± 0.07) than low-expression regions (0.05 ± 0.01, *P* < 0.001), indicating strong spatial agreement.** (D)** Sensitivity map analysis across 4,548 H&E WSIs, categorizing high-sensitivity regions by morphological feature: large-nucleated cells (diameter > 12 µm), cell clusters (≥ 5 nuclei), vascular-like structures, atypical cells, and bare nuclei (nuclear-cytoplasmic ratio > 0.8).** (E)** Hand-drawn schematic illustrating the five ecDNA-associated morphological feature categories identified by the model. **(F)** Validation on the 160-core CRC TMA; all five feature categories showed significantly elevated MYC fluorescence (> 7.5) compared to background (< 2.1), reinforcing the model's morphological basis.

We determined whether ecPICK could predict the spatial localization of ecDNA within tissues. DNA FISH was conducted on 160 colon cancer tissue samples using a *MYC* gene probe, a classic ecDNA-amplified oncogene, and the results were correlated with the ecPICK scores from the same samples. The ecPICK prediction score exhibited a significant positive correlation with the fluorescence signal intensity of the *MYC* gene (Pearson r = 0.92, R² = 0.85, *P* < 0.0001), indicating that ecPICK scores are strongly associated with the level of *MYC* amplification detected by FISH (Figure 5A). A FISH assay and corresponding ecPICK predictions were performed on a colon cancer cohort, which included adjacent normal tissues and Stage I and Stage III tumors (Figure 5B). As the clinical stage advanced, the intensity of the FISH signals increased concomitantly with increasing ecPICK scores.

Because of the difficulty in achieving single-cell resolution with the scores directly predicted by ecPICK, we used a Shapley Additive exPlanations sensitivity analysis to determine which morphological features in the H&E images are used by ecPICK for ecDNA prediction. This approach enables the evaluation of ecDNA-associated regions via cellular-level region-of-interest (ROI) feature attribution based on areas with high SHAP values. A co-localization analysis was performed between the high SHAP value regions and *MYC* amplification signals (defined as > 5 fluorescence signals per cell) in the 160 colon cancer tissue sections. As shown in Figure 5C, the results indicated an 89% spatial overlap rate between the *MYC* amplification areas and the model's high-attention regions (Jaccard index = 0.78). This indicates that ecPICK’s high-attention regions strongly overlap with areas of high *MYC* amplification—given that ecPICK was trained on AmpliconArchitect - defined ecDNA labels, these regions likely represent ecDNA-driven amplification in our study context.

To elucidate the specific morphological features in H&E images that ecPICK uses for ecDNA prediction, SHAP sensitivity analysis was performed to identify key morphological characteristics associated with high ecDNA probability regions (score > 0.8). Based on the TCGA pan-cancer cohort (n = 4,280) and 268 intrahepatic cholangiocarcinoma H&E whole-slide images (including adjacent normal tissues), the contribution of morphological features to the ecDNA prediction score was determined by calculating pixel-level SHAP values. The following cellular morphological regions significantly contributed to high ecPICK scores (SHAP value > 0.15, *P* < 0.001) (Figure 5D): Regions with large nuclei (nuclear diameter > 12 µm); Cell clusters; Vascular-like cells; Heterogeneous cells (areas with uneven nuclear chromatin contributed 4.2 times more than the baseline); and Bare nuclei (cytoplasm loss ratio > 40%). A schematic atlas illustrating these aforementioned five regions of cellular morphological features was generated (Figure 5E). Furthermore, the spatial comparison between H&E images containing these ecDNA-associated morphological features (enlarged nuclei, bare nuclei) and *MYC* FISH signals exhibited a high degree of overlap—supporting that these nuclear atypia are enriched in regions of high *MYC* amplification, aligning with our hypothesis that ecDNA-driven *MYC* overexpression drives the morphological signatures ecPICK detects.

### Spatial transcriptomics-based ecDNA prediction

**Figure 6:**
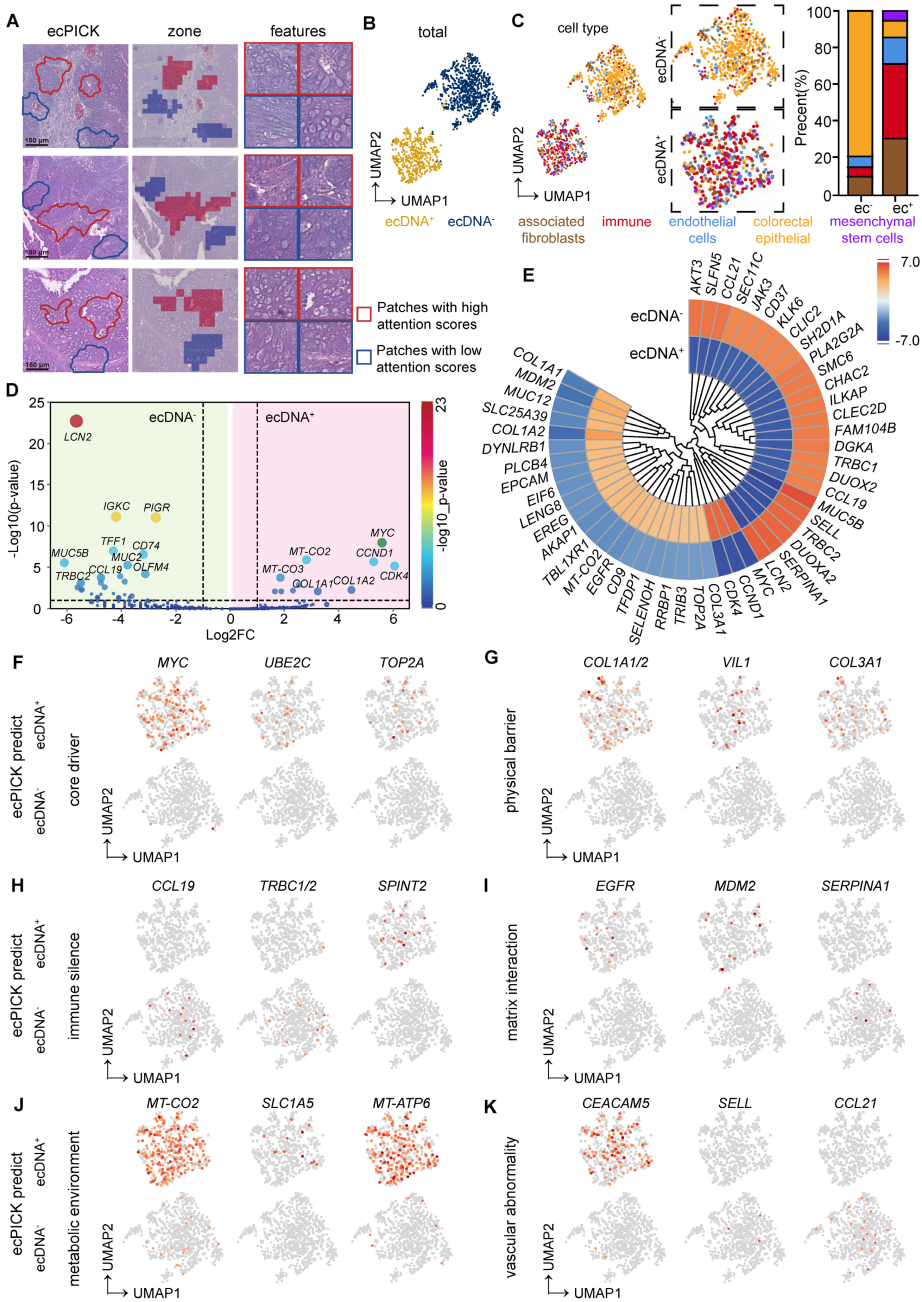
** Spatial transcriptomic analysis of ecDNA-positive and ecDNA-negative regions in colorectal cancer (CRC) samples. (A)** Regional annotation of nine CRC spatial transcriptomic samples (n = 160 regions). ecDNA-positive areas (score > 0.15) are marked red; ecDNA-negative areas (score < 0.1) are blue. Magnified views confirm enlarged nuclei and bare nuclei in ecDNA^+^ regions. **(B)** Spatial clustering separates cells from ecDNA^+^ and ecDNA^-^ areas.** (C)** Independent cell type clustering within the ecDNA-positive and ecDNA-negative groups. The ecDNA^+^ region composition: cancer-associated fibroblasts (30%), immune infiltrating cells (40%), colorectal cancer endothelial cells (15%), colorectal cancer epithelial cells (10%), and mesenchymal stem cells (5%). The ecDNA^-^ region composition: cancer-associated fibroblasts (10%), immune infiltrating cells (5%), colorectal cancer endothelial cells (5%), with the majority being colorectal cancer epithelial cells (80%). **(D)** Volcano plot of differential gene expression; significantly upregulated genes in ecDNA^+^ regions (|log2FC| > 2, -log10(P) > 5): *MYC* and *CCND1*; in ecDNA^-^ regions (|log2FC| > 2, -log10(P) > 10): *LCN2* and* CCL19*. **(E)** Circular clustered heatmap of transcriptomic differences; color intensity represents expression level; hierarchical clustering used complete linkage with Euclidean distance. **(F–K)** Differential expression across six functional dimensions: core dynamics **(F)**, physical barrier **(G)**, immune silencing **(H)**, stromal interaction **(I)**, metabolic environment **(J)**, and vascular abnormality **(K)**.

Because of the technical limitations in detecting ecDNA in cells, it has been challenging to examine the molecular characteristics of ecDNA-positive cells using methods such as single-cell sequencing. Exploiting ecPICK’s capability of predicting ecDNA localization from H&E-stained images enabled us to perform high-resolution spatial transcriptomic analysis on cancer tissues from nine patients with CRC, with a specific focus on the molecular differences between regions predicted by ecPICK as ecDNA-positive (ecDNA^+^) and ecDNA-negative (ecDNA^-^). Based on the results of the ecPICK sensitivity analysis, regions with a SHAP value > 0.15 (Figure 6A) were designated as ecDNA^+^, whereas those with a SHAP value < 0.05 were considered ecDNA^-^. The cell morphology in the ecDNA^+^ regions was consistent with the previously described features (enlarged nuclei, heterogeneity, and bare nuclei).

We performed a uniform manifold approximation and projection analysis on all selected regions (Figure 6B). The cells were separated into two groups, which were consistent with the ecPICK prediction for the ecDNA^+^ and ecDNA^-^ groups. The types of cells and their proportions were determined in the ecPICK-predicted ecDNA^+^ and ecDNA^-^ groups (Figure 6C). The ecDNA^+^ group consisted of five subpopulations: cancer-associated fibroblasts (CAFs, 30%), immune-infiltrating cells (40%), CRC endothelial cells (15%), CRC epithelial cells (10%), and mesenchymal stem cells (5%). In contrast, the ecDNA^-^ group primarily consisted of CRC epithelial cells (80%), with only small amounts of CAFs (10%), immune-infiltrating cells (5%), and CRC endothelial cells (5%). This clustering structure revealed that ecDNA^+^ regions have a more complex microenvironment, which suggests that ecDNA is a key factor in changing gene expression in tumor cells.

A differential gene expression analysis was conducted between the ecDNA^+^ and ecDNA^-^ groups. Volcano plot (Figure 6D) and clustered circular heatmap (Figure 6E) analyses revealed significantly upregulated genes (-log10(p-value) > 5) in the ecDNA^+^ group, which included *MYC*, *CCND1*, and *CDK4*, all of which are closely associated with malignant tumor proliferation. In the ecDNA^-^ group, significantly upregulated genes (-log10(p-value) > 10) included *LCN2*, *IGKC*, and *PIGR*.

The analysis revealed the following salient features within ecDNA-positive tissues:

Epigenetic Remodeling (Figure 6F): Characterized by aberrantly high *MYC* expression (Log2FC = 5.81), which drives ecDNA amplification; upregulation of *UBE2C* (Log2FC = 2.31), which mediates immune gene silencing through the ubiquitination pathway; and overexpression of *TOP2A* (Log2FC = 2.52), which sustains chromosomal instability.

Fibrotic Barrier Formation (Figure 6G): Mediated by significant upregulation of *COL1A1* (Log2FC = 3.30) and *COL1A2* (Log2FC = 3.91), which results in collagen deposition; enhanced cell-matrix adhesion facilitated by high *VIL1* (Log2FC = 2.25) expression; and synergistic action of *COL3A1* (Log2FC = 2.43), which contributes to extracellular matrix hardening.

Immunosuppressive Microenvironment (Figure 6H): The absence of the immune chemokine *CCL19* (Log2FC = -4.80) and the T-cell receptor genes *TRBC1/TRBC2* (Log2FC = -4.51), combined with the suppression of T/NK cell function mediated by the serine protease inhibitor *SPINT2* (Log2FC = 1.62), constitute an active immunosuppressive mechanism. As a result, although ecDNA^+^ cells accumulate a certain number of immune cells, their activity is suppressed.

CAF malignant cycle (Figure 6I): Driven by *EGFR* (Log2FC = 2.75) stimulating CAFs to secrete TGF-β; *MDM2* overexpression (Log2FC = 3.50) inhibits p53-dependent CAF apoptosis; and downregulates α1-antitrypsin *SERPINA1* (Log2FC = -5.66), thus reducing collagen degradation. These factors collectively result in hyperactivated CAFs, excessive collagen deposition, and the formation of a physical barrier, which fosters a vicious cycle that promotes tumor immune evasion and cancer cell proliferation.

Metabolic-Immune Uncoupled Escape (Figure 6J): This occurs through aerobic glycolysis (Warburg effect) facilitated by the mitochondrial genes *MT-CO2* (Log2FC = 2.72) and *MT-ATP6* (Log2FC = 2.11), resulting in elevated mitochondrial metabolism and lactate accumulation, along with glutamine (Gln) depletion attributed to the increased expression of the glutamine transporter *SLC1A5* (Log2FC = 1.75). These metabolic changes collectively inhibit T-cell-mediated immune responses.

Aberrant Vasculature Further Impeding Immune Infiltration (Figure 6K): *CEACAM5* (Log2FC = 2.30) promotes vascular leakage, whereas the loss of L-selectin *SELL* (Log2FC = -5.51) inhibits immune cell adhesion, which collectively disrupts vascular endothelial barrier function. Decreased *CCL21* (Log2FC = -5.15) prevents the formation of high endothelial venule (HEV)-like vessels, which results in a lack of endothelial activation and disorganized vascular networks, ultimately leading to the creation of an “immune desert” that suppresses T-cell infiltration.

These results indicate that ecDNA-positive regions act as treatment-resistant cold niches defined by physical collagen barriers and immune silencing. The observed mitochondrial metabolic reprogramming (e.g., *MT-CO2*, *MT-ATP6*) indicates that ecPICK can identify specific tumor areas that may benefit from combined metabolic inhibitors and ecDNA-targeted therapies. Thus, ecPICK is not only a detection tool, but also a guide for customizing localized therapeutic interventions.

To further elucidate the spatial distribution patterns of the aforementioned genes within tissues, their localization was validated (Figure S2). In addition, KEGG and GSEA pathway enrichment analyses (Figure S3) provided further evidence supporting the aforementioned conclusions.

## Discussion

In this study, we developed a deep learning model, ecPICK, which uses routinely acquired H&E-stained WSIs to directly predict the abundance and spatial localization of ecDNA. As an end-to-end model that operates without relying on additional experimental techniques, such as FISH or WGS, ecPICK has substantial potential and represents a paradigm shift by markedly lowering the barrier for clinical ecDNA detection. This tool provides a powerful resource for basic research, clinical applications, and translational studies in the ecDNA field. Our subsequent clinical analysis of the model-predicted ecDNA scores revealed significant correlations with patient outcomes, including overall survival, clinical stage, and histological grade. This indicates that ecDNA may serve as a novel cancer diagnostic and prognostic biomarker. Consequently, ecPICK may enhance tumor risk stratification and guide therapeutic decision-making. Using ecPICK, we identified distinctive cytomorphological features associated with ecDNA-positive cells, such as enlarged nuclei (diameter > 12 µm), high nuclear heterogeneity, bare nuclei, cell clusters, and vascular-like cells. Furthermore, by integrating SHAP analysis with spatial transcriptomics, we identified distinct gene expression signatures in ecDNA-positive regions.

This study established, for the first time, an end-to-end neural network that directly maps H&E images to ecDNA scores. Compared with other approaches, such as the ecPath model 34 proposed by Mudra Choudhury et al., ecPICK has fundamental differences and advantages: (1) Model Architecture: Although ecPath indirectly infers ecDNA status by first predicting transcriptomic data from H&E images and then applying traditional machine learning, ecPICK utilizes a true end-to-end deep learning architecture. It directly learns the association between morphological features in H&E images and the presence of ecDNA, thereby avoiding the transcriptional noise and uncertainties associated with cross-omics integration. (2) Generalizability and Applicability: ecPath was separately built on limited cancer types, whereas ecPICK was trained on a pan-cancer TCGA dataset comprising 20 cancer types. This enables ecPICK to demonstrate stable performance across different tissues and cancer lineages, thus enhancing its clinical utility. (3) Interpretability and Biological Insight: ecPath primarily offers explanations at the gene expression level. In contrast, ecPICK systematically links ecDNA status directly to cellular morphological features, which fills an important gap in understanding the tissue-level phenotypic manifestations of ecDNA.

The clinical predictions produced by ecPICK are highly congruent with the findings from previous studies. Our results indicate a significant correlation between ecDNA and adverse prognosis, with ecDNA-positive cases being more prevalent among older patients (e.g., > 60 years), in patients with high-grade tumors (Stage III–IV), and patients who have undergone chemotherapy or radiotherapy. These cases were associated with reduced survival, and the observations are consistent with and reinforce conclusions from studies that identified ecDNA using other techniques, such as WGS or FISH 1, 3, 4, 7, 35. Moreover, the high-resolution spatial transcriptomic analysis of ecDNA in the present study aligns closely with the previously described biological functions of ecDNA. The essential roles of ecDNA in enhancing mitochondrial metabolic activity, inducing immunosuppression, and facilitating the remodeling of the tumor microenvironment 36-39 are strongly supported by our spatial analysis results. They validate the accuracy and reliability of the ecPICK predictive model from various perspectives.

The development of ecPICK is beneficial for basic ecDNA studies and clinical translation. Regarding basic research, it provides scientists with a powerful, low-cost method to determine the status of ecDNA directly from tissues at spatial resolution, making it easier to conduct large-scale studies while avoiding complicated experimental procedures. The morphological associations provide researchers with new ideas regarding how ecDNA affects nuclear structure and function. For clinical applications, the model represents an easy, quick, and inexpensive way to identify ecDNA with only standard H&E slides. This makes it easier to use ecDNA as a novel prognostic or predictive biomarker. Unlike binary classification, our three-tier strategy considers the natural biological heterogeneity and morphological ambiguity of ecDNA among various cancers. Almost half of the population was successfully filtered out with 95% specificity and sensitivity, and ecPICK significantly reduced the workload and costs associated with gold-standard assays, such as FISH or WGS. Methodologically, ecPICK represents the successful application of “morphomics” in tumor molecular subtyping. It establishes a paradigm for extracting molecular phenotypes from conventional pathology images and broadening the scope of artificial intelligence in digital pathology.

The ICC validation cohort exhibited substantial domain shifts compared with the TCGA training set, particularly with respect to ethnicity (East Asian versus predominantly Western populations) and technical variability in tissue processing and digital scanning. The strong performance (AUROC = 0.913, 95% CI: 0.898–0.927, Figure S1) across these geographical and technical boundaries highlights the significant generalizability of ecPICK and its potential for applications in various clinical contexts. Because the morphological signature of ecDNA on H&E was previously undefined, traditional visual validation by pathologists cannot currently serve as a ground truth. Therefore, our study uniquely leverages spatial FISH validation (Figure 5) to quantitatively anchor these deep learning-derived morphological features to a definitive molecular reality. This pipeline not only deciphers the “black box” of our model, but also provides the pathologists with the first visual lexicon for ecDNA-driven malignancies.

ecPICK represents a substantial paradigm shift in precision diagnostics. By integrating spatial genomic information into the standard pathological workflow, it functions as a digital companion diagnostic (dCDx) to evaluate the genetic drivers and metabolic landscape of tumors. This “theranostic” capability is important for identifying patients at high risk of relapse and designing personalized treatment strategies in ecDNA-driven malignancies.

There is room for further optimization and deeper analysis of ecPICK. The current model predicts overall ecDNA abundance, but it cannot precisely identify specific amplified genes or structural variations on ecDNA. Moreover, the model’s accuracy can be further improved, and its current application is limited to H&E-stained images. Future studies should explore integrating information from other stains, such as DAPI (4', 6-diamidino-2-phenylindole). It will also be important to leverage ecPICK as a companion diagnostic tool to rapidly screen patients for targeted therapy clinical trials, potentially improving success rates and drug response. Furthermore, it may be used to monitor dynamic changes in ecDNA load during treatment, revealing the evolution of resistant clones and providing a rationale for designing drugs that target ecDNA stability or combination strategies (e.g., with DDR inhibitors or epigenetic drugs).

## Supplementary Material

Supplementary figures and materials and methods.

## Abbreviations

ecDNA: Extrachromosomal DNA
H&E: Hematoxylin and Eosin
WSI: Whole-Slide Image
FISH: Fluorescence *In Situ* Hybridization
AUC/AUROC: Area Under the (Receiver Operating Characteristic) Curve
WGS: Whole-Genome Sequencing
ResNet: Residual Network
DNN: Deep Neural Network
GAP: Global Average Pooling
SHAP: SHapley Additive exPlanations
Grad-CAM: Gradient-weighted Class Activation Mapping
TCGA: The Cancer Genome Atlas
ICC: Intrahepatic Cholangiocarcinoma
