# Supplementary material for: ecPICK: A deep learning-enabled spatial diagnostic platform for direct ecDNA identification and clinical prognosis across pan-cancer histopathology
Source: Theranostics. 2026 May 25;16(12):7067–83. doi: 10.7150/thno.134316 (PMC13232621; doi:10.7150/thno.134316)
Supplement: Supplementary file 1 — Supplementary figures and materials and methods. [file thnov16p7067s1.pdf]

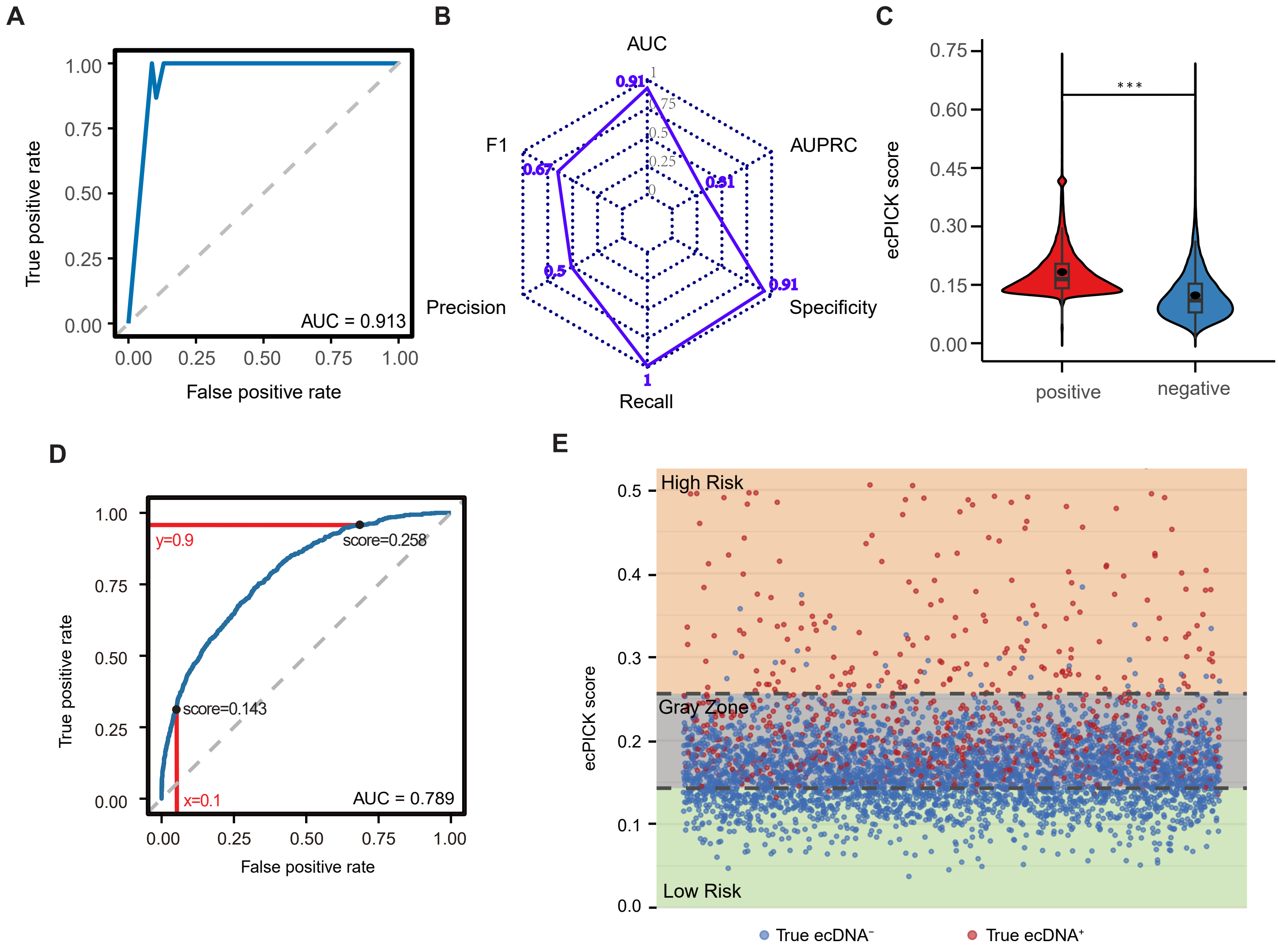

F

	Score	Metrics	Decision	Cohort%
High Risk	＞0.258	Rule-in<br>High Specificity	Identification	17.6%
Gray Zone	0.143-0.258	Indeterminate	Validation	50.6%
Low Risk	＜0.143	Rule-out<br>High Sensitivity	Exclusion	31.8%

Figure S1. Validation of ecPICK in an independent ICC cohort and establishment of the clinical subcategory threshold.

(A) ROC curve on 268 ICC H&E WSIs; AUC = 0.913 (95% CI: 0.887–0.939). **(B)** Radar chart of six metrics in the validation cohort: AUC = 0.91, AUPRC = 0.51, F1-score = 0.67, Precision = 0.50, Recall = 1.00, Specificity = 0.91. **(C)** Violin plot showing higher median prediction score in ecDNA-positive cases (0.17, IQR: 0.14–0.18) than ecDNA-negative cases (0.10, IQR: 0.08–0.15; Mann–Whitney $P < 0.001$, Cohen's d = 2.1). **(D)** Threshold selection on pan-cancer ROC: rule-out bound at 0.143 (High sensitivity); rule-in bound at 0.258 (High specificity). **(E)** Three-tier stratification of TCGA cohort (n = 4,280): Low Risk (green), Gray Zone (gray), High Risk (orange); 31.8% classified as low-risk at high sensitivity. **(F)** Summary table of score ranges, operational targets, and recommended clinical actions per tier; Gray Zone (50.6%) prioritizes WGS or FISH validation.

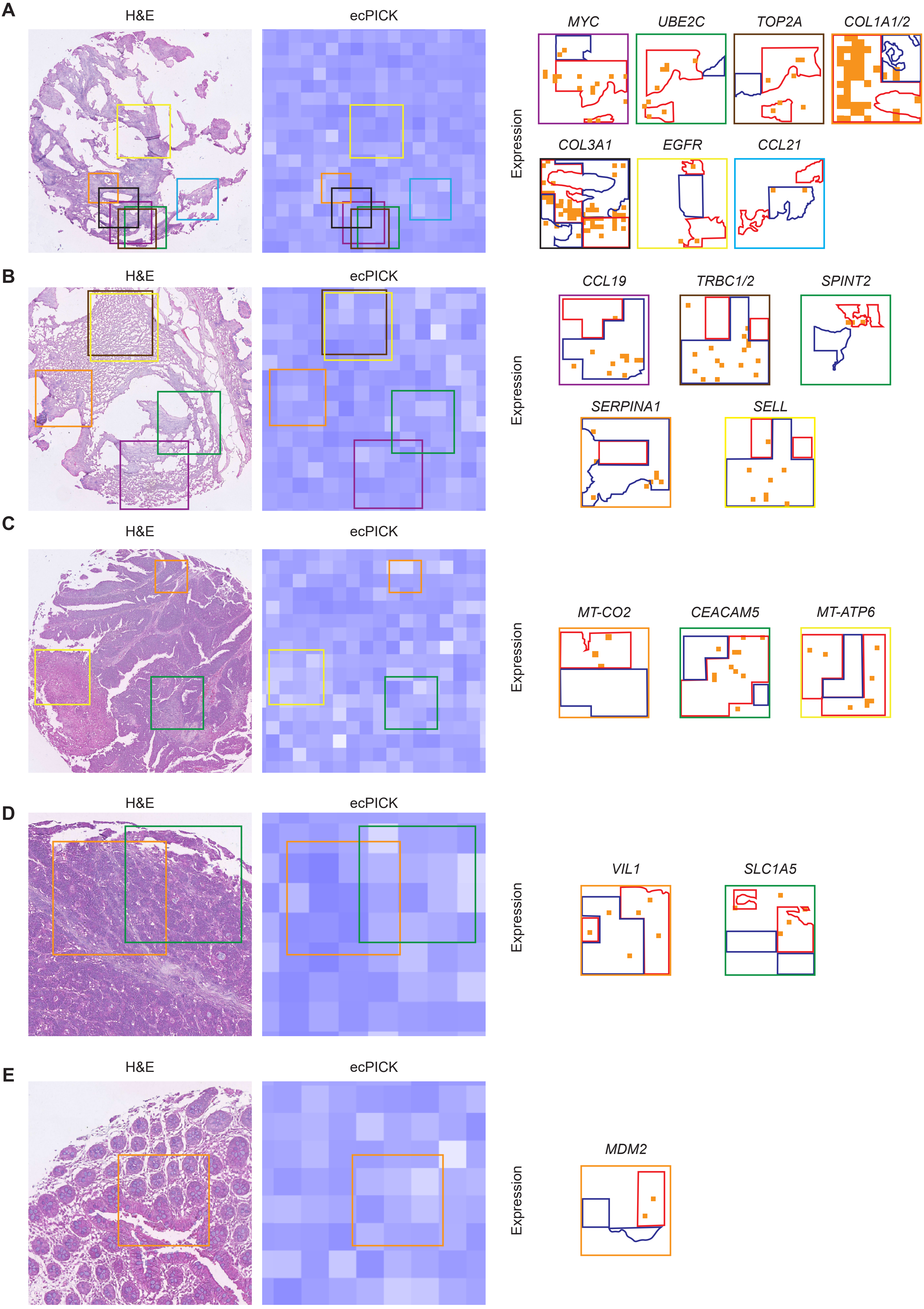
A
H&E
ecPICK
Expression
MYC
UBE2C
TOP2A
COL1A1/2
COL3A1
EGFR
CCL21
B
H&E
ecPICK
Expression
CCL19
TRBC1/2
SPINT2
SERPINA1
SELL
C
H&E
ecPICK
Expression
MT-CO2
CEACAM5
MT-ATP6
D
H&E
ecPICK
Expression
VIL1
SLC1A5
E
H&E
ecPICK
Expression
MDM2

Figure S2. Spatial transcriptomic mapping of gene expression in ecDNA-positive and ecDNA-negative regions defined by ecPICK.

(A-E) Spatial expression profiles of representative genes across colorectal cancer tissue microarray cores. Each gene is displayed in selected regions of the H&E staining. In the "expression" panel, the red box represents the model-predicted ecDNA^{+} region, the blue box represents the model-predicted ecDNA^{-} region, and the yellow dots indicate the gene's expression locations within these regions. **(A)** Spatial distribution of *MYC*, *UBE2C*, *TOP2A*, *COL1A1/2*, *COL3A1*, *EGFR*, and *CCL21* within the same H&E-stained tissue region from a single patient. **(B)** Spatial expression of *CCL19*, *TRBC1/2*, *SPINT2*, *SERPINA1*, and *SELL* mapped onto a shared H&E region from another patient. **(C)** Expression patterns of *MT-CO2*, *CEACAM5*, and *MT-ATP6* within the same H&E-stained tissue region from a single patient. **(D)** Spatial mapping of *VIL1* and *SLC1A5* within the same H&E-stained tissue region from a single patient. **(E)** Spatial expression of *MDM2* in the corresponding H&E section of another patient.

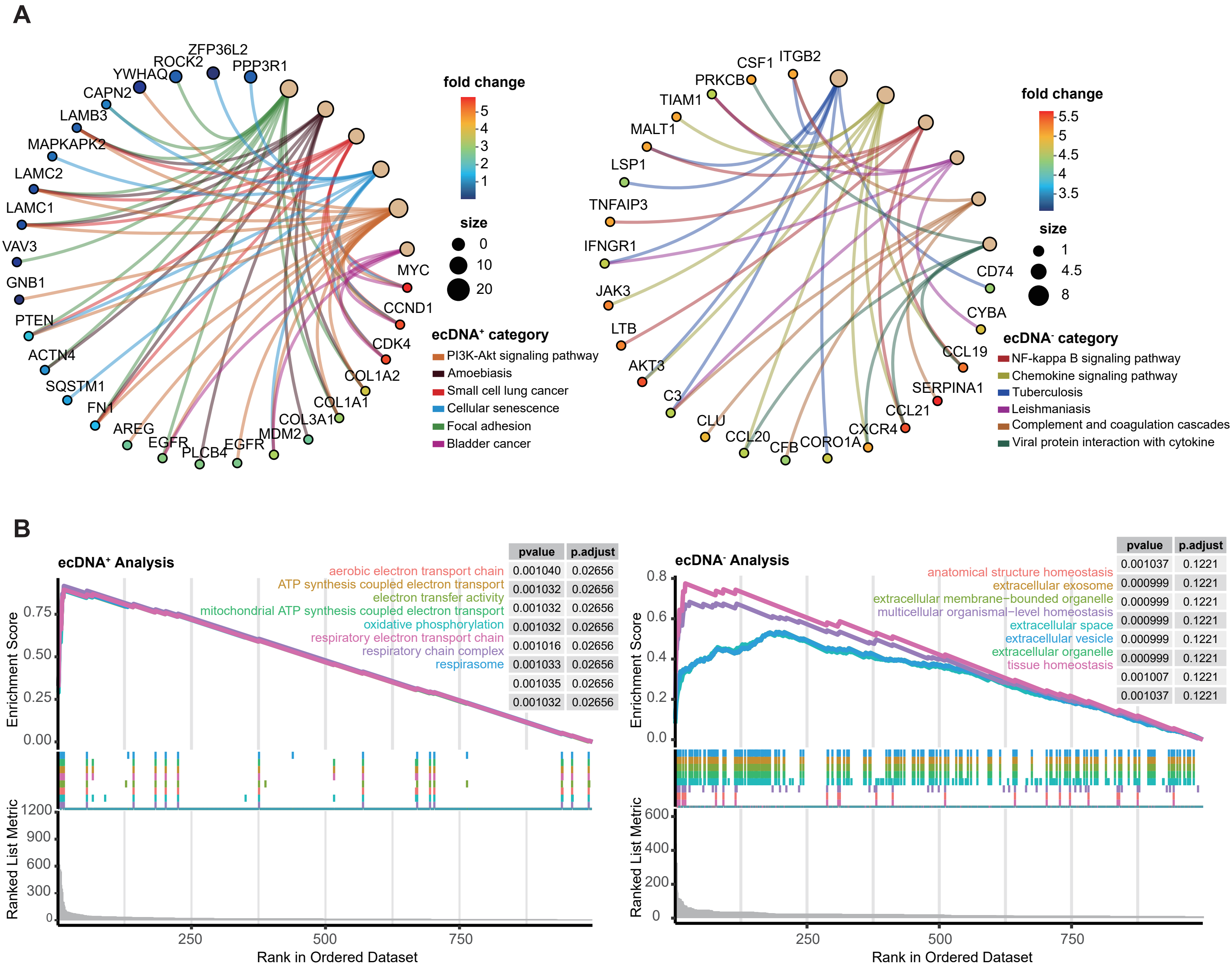

	pvalue	p.adjust
aerobic electron transport chain	0.001040	0.02656
ATP synthesis coupled electron transport	0.001032	0.02656
electron transfer activity	0.001032	0.02656
mitochondrial ATP synthesis coupled electron transport	0.001032	0.02656
oxidative phosphorylation	0.001016	0.02656
respiratory electron transport chain	0.001033	0.02656
respiratory chain complex	0.001035	0.02656
respirasome	0.001032	0.02656

	pvalue	p.adjust
anatomical structure homeostasis	0.001037	0.1221
extracellular exosome	0.000999	0.1221
extracellular membrane−bounded organelle	0.000999	0.1221
multicellular organismal−level homeostasis	0.000999	0.1221
extracellular space	0.000999	0.1221
extracellular vesicle	0.000999	0.1221
extracellular organelle	0.001007	0.1221
tissue homeostasis	0.001037	0.1221

Figure S3. KEGG and GSEA analyses reveal distinct pathway activities in ecDNA-positive and ecDNA-negative regions.

(A) KEGG pathway enrichment results. **(B)** GSEA pathway enrichment results.

Supplementary Methods

Model Training Data

(1) Public Cohort：

H&E-stained WSIs were obtained from TCGA using the TCGAbiolinks R package. The inclusion criteria comprised primary tumors with a tumor nuclei proportion ≥ 50%, necrosis ≤ 20%, and slides scanned at 40× magnification (0.25 μm/pixel). A total of 20 cancer types were included, with FFPE sections constituting the training set. From these slides, 4,280 image-level predictions were generated. Data usage complied with TCGA data access policies, and no additional ethical approval was required.

(2) Internal Cohort:

The internal cohort consisted of 134 postoperative samples from ICC patients at Tianjin Medical University Cancer Hospital. For each case, two representative FFPE block was selected, sectioned at 4 μm thickness, and stained with H&E. Slides were digitized using an Aperio GT450 whole-slide scanner at 40× magnification (0.22 μm/pixel), producing uncompressed TIFF files (RGB, 8-bit). DNA extracted from the same tissue blocks underwent 30× whole-genome sequencing (WGS). The H&E WSIs and corresponding patient information formed an independent validation cohort for the model, with ecDNA status determined by AmpliconArchitect v1.2[1]. Additionally, a prospective study cohort was established using tissue microarrays (TMAs) purchased from the tissue bank of Changsha Yaxiang Biotechnology Co., Ltd., comprising 80 CRC and 75 HCC cases. Ultimately, TMA core images were obtained from 150 HCC and 160 CRC cases, including both cancerous and adjacent normal tissues. All slide images were saved in uncompressed TIFF format and underwent color normalization using Macenko's method[2-4] to minimize staining batch effects and ensure data consistency.

ecDNA Clustering Analysis

We employed the AmpliconArchitect tool, as described by Roel G. W. Verhaak et al.[1] , to analyze WGS data from patients in TCGA and 134 ICC patients from Tianjin Medical University Cancer Hospital. The specific analytical workflow was as follows: First, the sequencing reads were aligned to the hg38 reference genome using BWA-MEM (v0.7.17). Somatic copy number variations (CNVs) were then detected using GATK (v4.2.0). Subsequently, AmpliconArchitect was executed to identify

ecDNA structures (parameters: minCN = 4, minSize = 10kb). Finally, based on the presence or absence of ecDNA, samples were categorized into ecDNA-positive ($ecDNA^+$) and ecDNA-negative ($ecDNA^-$) groups.

Tissue DNA FISH Combined with H&E Staining

TMA sections of 4 μm thickness were baked in an oven at 85 °C for 10 minutes to enhance tissue adhesion and remove residual moisture. After cooling to room temperature, pretreatment was conducted according to the instructions of the EXONBIO Cell Sample FISH Pretreatment Kit (Cat# D-0014), using the pretreatment reagent at 37 °C for 20 minutes, followed by rinsing with 1 × wash buffer and dehydration through an ethanol gradient (70%, 85%, 100%, 2 minutes each). For each tissue core, 10 μl of the *MYC* DNA FISH probe from FOCOFISH (Cat# FD-1023H) was applied, covered with a 22 × 22 mm coverslip, and the edges were sealed with FISH mounting glue. Denaturation was performed on a metal bath at 85 °C for 4 minutes, followed by hybridization in a humidified chamber at 42 ℃ for 17 hours. Post-hybridization, the coverslips were removed, and the slides were washed in 1 × wash buffer at 74 °C for 2 minutes, followed by a final wash in 1 × wash buffer at room temperature for 5 minutes. Finally, counterstaining was performed using an antifade mounting medium containing 10 μg/ml DAPI for 5 minutes before sealing.

Fluorescence images were acquired using a Pannoramic MIDI II slide scanner (3DHISTECH, 40× /0.95 NA objective, 385 nm and 560 nm filters, pixel size 0.32 μm). Immediately after scanning, the coverslips were gently removed, and the same sections were subjected to sequential H&E staining to ensure precise correspondence between FISH signals and histological regions. The sections were stained in hematoxylin solution for 4 minutes, rinsed under running water for bluing; if necessary, differentiation was performed in 1% acid alcohol, followed by bluing in warm water (37 °C) for 30 seconds. Staining in eosin solution (diluted 1:2 with distilled water) was carried out for 35 seconds, followed by a gentle rinse under running water. Dehydration was performed through a graded ethanol series (70%, 80%, 95%, 100%, 30 seconds each), followed by clearing in xylene twice (3 minutes each) and mounting with neutral balsam. The paired FISH and H&E images were subsequently aligned at the pixel level using a rigid registration script to enable precise correlation between ecDNA signals and tissue morphology.

Image Analysis

Quantification of fluorescence intensity distribution was performed using the

Profile and Measure functions within ZEN Blue software (Zeiss). The Analyze Particles module in ImageJ software (NIH) was employed to analyze the number, area, and foci count of fluorescent signal points. Three-dimensional reconstruction of fluorescent signals was conducted using Imaris 9.5 (Oxford Instruments). A threshold of ⩾ 5 focal signals per cell was used as the criterion for defining positivity.

Clinical and Prognostic Analysis Methods

(1) Survival Analysis

Overall survival (OS) was compared between high and low ecDNA score groups using Kaplan-Meier curves and a two-sided log-rank test. Variables including ecDNA content, age, sex, TNM stage, and treatment status were initially screened using univariate Cox models and subsequently included in Chi-square tests to quantify their association strength with 3-year and 5-year survival rates. The Mann-Whitney U test was used for comparing non-normally distributed continuous variables between groups, while categorical clinical variables were analyzed using the Chi-square test or Fisher's exact probability method. All statistical analyses were performed in R 4.3.0, with a significance threshold set at a two-sided $P < 0.05$ and multiple comparisons adjusted using the Benjamini-Hochberg method.

(2) Clinical Feature Correlation Analysis

The Mann-Whitney U test was applied to assess correlations between the ecDNA score and ordinal or continuous clinical parameters, such as AJCC 8th edition staging, response to radiotherapy/chemotherapy (based on RECIST 1.1 criteria), and age. Results were visualized using bubble plots (effect size vs. significance) and box plots (median ± IQR). To validate the independent prognostic value of the ecDNA score, a bidirectional stepwise Cox proportional hazards regression model was constructed (entry $\alpha = 0.05$, removal $\alpha = 0.10$), controlling for variables including TNM stage (ordinal I-IV), grade (ordinal I-III), stage (ordinal I-IV), and preoperative treatment status (radiotherapy/chemotherapy). The proportional hazards assumption was tested using Schoenfeld residuals; when the assumption was violated, time-dependent covariates were introduced. The final model reported hazard ratios (HR) with 95% confidence intervals. Model stability was assessed through 1,000 bootstrap internal validation and 10-fold cross-validation.

Analysis of Gene Expression Features in ecDNA-Positive/Negative Regions

(1) Spatial Transcriptomic Analysis

Spatial transcriptomic analysis of colorectal cancer tissues from nine patients

was performed using the 10x Genomics Visium HD platform. Based on ecPICK-derived SHAP values, spots were categorized as ecDNA-positive (SHAP > 0.15) or ecDNA-negative (SHAP < 0.05). These regions were manually annotated in Loupe Browser 8.0. Differential expression analysis was conducted with DESeq2 (v1.38), using a model that incorporated patient source as a blocking variable. Significantly differentially expressed genes (FDR-adjusted $\alpha = 0.05$) were identified. Data visualization utilized UMAP (parameters: minimum distance = 0.5, n_neighbors = 10). Functional comparison of upregulated gene modules between regions was performed using the compareCluster function from the clusterProfiler R package.

(2) Gene Enrichment and Pathway Analysis

To systematically interpret the functional annotations, associated biological pathways, and regulatory relationships of the differentially expressed genes (DEGs) identified between $ecDNA^+$ and $ecDNA^-$ regions in the spatial transcriptomic data, both KEGG pathway enrichment analysis and Gene Set Enrichment Analysis (GSEA) were performed. The DEG list (filtered by $|log2FC| > 0.5$ and $FDR < 0.05$) was converted to Ensembl IDs and used as input for the clusterProfiler package (v4.8). KEGG pathway enrichment was carried out (organism = "hsa", p.adjustCutoff = 0.05, qvalueCutoff = 0.2), with a focus on pathways such as PI3K-Akt, MAPK signaling, Cell Cycle, and Purine metabolism. Concurrently, GSEA was executed using the fgseapackage (v1.24) with 10,000 permutations. Gene sets were sourced from MSigDB Hallmark and GO-BP (release 2023-1), and phenotypes were ranked based on the Signal-to-Noise Ratio (SNR). For immune microenvironment characterization, the IOBR package was additionally used to estimate the relative abundance of 28 immune cell types. Spearman's correlation test was then applied to examine the association between the ecDNA score and the activity of metabolism-immune cross-talk pathways. For all enrichment results, a normalized enrichment score ($|NES|$) > 1.5 combined with an FDR < 0.25 served as the significance threshold. The robustness of these findings was further validated through 1,000 permutation tests.

Reference

1. Kim H, Nguyen NP, Turner K, Wu S, Gujar AD, Luebeck J, et al. Extrachromosomal DNA is associated with oncogene amplification and poor outcome across multiple cancers. Nat Genet. 2020; 52: 891-7.
2. Kather JN, Heij LR, Grabsch HI, Loeffler C, Echle A, Muti HS, et al. Pan-cancer image-based detection of clinically actionable genetic alterations. Nat Cancer. 2020; 1: 789-99.
3. Benjamini D, Iacono D, Komlosh ME, Perl DP, Brody DL, Basser PJ. Diffuse axonal injury has a characteristic multidimensional MRI signature in the human brain. Brain. 2021; 144: 800-16.
4. Bai B, Yang X, Li Y, Zhang Y, Pillar N, Ozcan A. Deep learning-enabled virtual histological staining of biological samples. Light Sci Appl. 2023; 12: 57.
